# Innovating workplace learning: Training methodology analysis based on content, instructional design, programmed learning, and recommendation framework

**DOI:** 10.3389/fpsyg.2022.870574

**Published:** 2022-07-22

**Authors:** Sehoon Kim

**Affiliations:** ^1^Seoul School of Integrated Sciences and Technologies, Seoul, South Korea; ^2^Business School Lausanne, Chavannes-près-Renens, Switzerland; ^3^The Institute for Industrial Policy Studies, Seoul, South Korea

**Keywords:** COVID-19 training, training effectiveness, workforce upskilling, technology-enhanced training, CIP-R framework, CIPP model, digital training, hybrid learning

## Abstract

The quality of sales processes is crucial in automotive and directly related to the firm’s competitive advantage and financial success. Sales training is the most prevalent intervention to guarantee quality and productivity. Extant literature has attempted to measure training effectiveness adequately, and the Context, Input, Process, and Product evaluation (CIPP) model has been a popular approach. This study endeavored to advance current literature and suggest a novel effectiveness framework, Content, Instructional design, Programmed learning, and Recommendation (CIP-R). The framework was applied to examine three different methodologies—traditional, pure digital, and hybrid training—collecting 583 instances from the automotive sales training conducted from 2019 to 2020 in South Korea. The findings advocate the importance of human elements, the role of efficacy, and self-determination in generating learning transferability, leading to performance in the digital age.

## Introduction

Coronavirus disease 2019 (COVID-19) created a global mechanism that “forced” online learning to be a reasonable alternative to traditional ways of developing people (Lands and Pasha, 2021). HR managers began to rethink development initiatives, providing flexible, personalized online learning opportunities (Li and Lalani, 2020). Training online could comfort the employees, reducing their skill gaps (Zhang et al., 2018) under isolated situations, and global institutions started providing digital workforce development and upskilling programs (Lands and Pasha, 2021).

A skilled workforce is substantial when it comes to the sales function. A firm’s economic success depends on the relationships between the customers and the employees that function as the interface that connects the company’s value chain to the market (Davenport, 2012). Ever-elevating customer expectations upon better services and products, complex buying and selling processes would draw more pressure and let businesses pay attention to the quality and competencies of their sales organizations (Zoltners et al., 2008; Williams et al., 2017; Arli et al., 2018; Hartmann et al., 2018; Hartmann and Lussier, 2020; Lim, 2020). The automotive industry is the largest sector in overall retail spending (Palandrani, 2020) and a vital customer that plays a crucial role in the industry ecosystem (Mathur and Kidambi, 2012; Dweiri et al., 2016). The quality of sales processes is crucial in auto retail since that is where the customers perceive the brand’s quality (Hoffmeister, 2018). Hence, automakers are expected to focus on human resource development (HRD) in retailing. Sales training is the most prevalent and widespread intervention to guarantee quality and increase productivity (Singh and Venugopal, 2015; Kodwani and Prashar, 2019). Today’s sales organizations proactively adopt digitalized training to enhance efficiency and effectiveness (Zoltners et al., 2021). However, it remains uncertain to determine the most optimized way of adopting technology in sales training.

Maximizing the effectiveness is the primary goal for any training initiative (Sitzmann and Weinhardt, 2015) and measuring the outcomes is essential to ensure the continuation and improvement of interventions (Kirkpatrick and Kirkpatrick, 2006). The Context, Input, Process, and Product evaluation (*CIPP*) model is one of the most frequently used frameworks (Adedokun-Shittu and Shittu, 2013; Finney, 2019). However, its limitations, such as time, cost, complexity, and lack of the participants’ voice, hinder practitioners and researchers from adopting the framework despite its advantages (e.g., Robinson, 2002; Tan et al., 2010). Furthermore, defining the training *effectiveness*, which includes learning transfer and post-training changes, is vital in the existing training literature (Alvarez et al., 2004). Hence, the CIPP model requires further theoretical review to prove itself as a robust and valuable framework (Finney, 2019). In the post-COVID era, more businesses are expected to provide entirely digital or hybrid learning opportunities for their workers, replacing traditional classrooms. It is required to empirically compare and analyze the effectiveness of traditional and technology-enhanced training. The CIPP model requires further effort to reflect the changing trend and theoretical attempt to calculate the “effectiveness” over “evaluation.”

From the abovementioned data, the study seeks to propose a new framework based on the CIPP model, advancing it into a practical, trainee-centered model. Based on the new framework, the study aims to compare the outcomes of three methodologies, namely, *traditional, pure digital, and hybrid training* and measure the effectiveness of each method. The study then seeks to analyze the factors that generated the differences in the training outcome. In this respect, the study presents the following research questions: (1) What is the optimal way of measuring training effectiveness in the digital age? (2) How does the training effectiveness change as digital technologies involve? and (3) What are the factors that affect training effectiveness?

This article is structured as follows. Section 2 provides theoretical background and a literature review related to the research. Section 3 deals with the methodology used in the study, analytical procedure, and details for data collection. Section 4 addresses the study’s results and Section 5 summarizes and concludes the research findings. Section 6 proposes theoretical and practical implications. Finally, Section 7 presents limitations and suggestions for future researchers.

## Theoretical background

### Training of sales organization

Modern consumers expect extended service and consumer-centric behaviors from salespeople (Lassk et al., 2012). Businesses focus on keeping the quality of the sales by investing in training (Kodwani and Prashar, 2019). Firms strive to meet consumers’ escalating expectations and manage organizational resources to provide quality service (Shabani et al., 2017). Managers expect the training to result in several returns, such as salesforce motivation and sales competence (Kauffeld and Lehmann-Willenbrock, 2010; Panagopoulos et al., 2020), leading to overall performance enhancement and goal achievement (Salas-Vallina et al., 2020). Sales training is the most frequent and universal intervention to improve sales productivity or customer orientation (Singh and Venugopal, 2015; Kodwani and Prashar, 2019). Effective sales training interventions may enhance sales organizations’ knowledge, skill, and performance (Lichtenthal and Tellefsen, 2001; Singh et al., 2015).

The most common challenges that organizations undergo are (1) constant changes in salespeople’s role and definition, (2) emphasis on accountability and reliability of sales function, and (3) reskilling and upskilling of salesforce facing new technologies (Lassk et al., 2012). However, one of the less explored issues is the (1) understanding of differences in training methodologies and (2) measurement for training evaluation and effectiveness (e.g., Lupton et al., 1999; Attia et al., 2021). This article discusses two primary challenges in the following sections of the literature review, namely, training methodologies and the training evaluation/effectiveness measurements.

### Digital technology in a corporate learning context

Using technology in training must conform to the changing environment (Hartmann and Lussier, 2020). Digital transformation of salesforce pursued by firms has been accelerating (Guenzi and Nijssen, 2021) due to the merits, such as elevated efficiency and effectiveness (Zoltners et al., 2021). Online learning is now replacing traditional, instructor-led, and offline settings, becoming a significant corporate learning and development element to upskill employees based on a lower cost (Lands and Pasha, 2021). Advanced technologies adopted in the learning management system provide a flexible learning environment (Traxler, 2018), and technologies such as gamified learning could affect users’ flow and continuous usage (Kim, 2021).

However, a single method (purely online or purely offline) might be challenging to satisfy all learners. Instead, by combining online and offline, learning satisfaction could be increased (Mantyla, 2001). Integrating technology with traditional methods has raised the interest in *“Blended learning”* (Hrastinski, 2019). *Blended learning* combines traditional methods with technology-based learning and avoids the demerits of offline and online learning (e.g., Driscoll, 2002; Graham, 2006). At present, the terms blended learning and hybrid learning are interchangeably used (Watson, 2008; Graham, 2009) and are regarded as one of the most suitable modes for training in the digital era (Liu et al., 2020). Consequently, technology-blended learning could lead to best practices for businesses (Singh and Reed, 2001; Driscoll, 2002) since the learners’ cognitive process and the training/teaching strategy might change (Liu et al., 2020).

**FIGURE 1:**
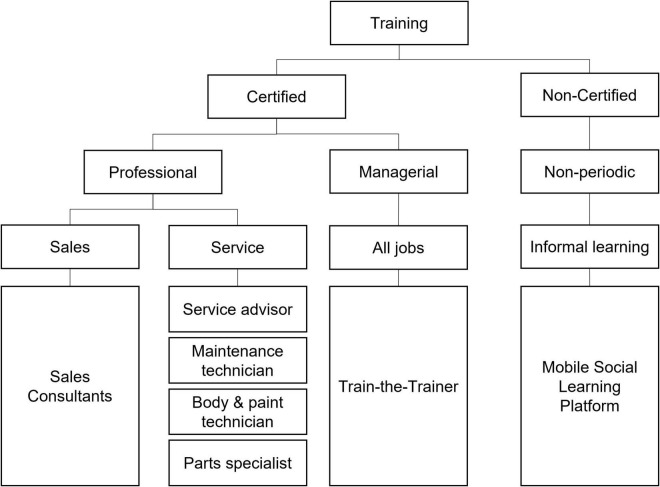
Human resource development at Company A.

This study takes the empirical case from a sales training program conducted by Company A in South Korea. The firm is the regional headquarters of a multinational automotive company. The firm’s overall HRD system is shown in Figure 1. The sales training program, which is the main interest of this study, is named *sales consultant training and education program* (STEP). The company had difficulties conducting face-to-face sales training during COVID-19. Consequently, the firm decided to implement salesforce training using digital technology. In this respect, two alternatives, namely, structured pure online training and hybrid training that mixed offline and digital, were developed and applied.

### Training transfer, evaluation, and effectiveness

Measurement of training outcome contributes to the continuity of training and program improvement (Kirkpatrick and Kirkpatrick, 2006). *Training evaluation* is an act of evaluating the value or quality of training elements systematically and scientifically. The expectancy theory by Vroom (1964) supports the views of prior researchers that the training’s perceived value or trainees’ beliefs (valence) about the training outcome would be crucial to training success (Colquitt et al., 2000). If the trainees get motivated, their job performance may increase with the feeling of accomplishment, which may also provide the potential for their future growth (e.g., Hanaysha and Tahir, 2016; Ibrahim et al., 2017; Wolor et al., 2020).

One of the most widely accepted performance indicators is the *transfer performance of learning* (Leach and Liu, 2003). Changes might occur when the employees transfer what was learned to their workplace (i.e., Ployhart and Hale, 2014). Researchers have insisted that training efforts will not bring the desired outcomes if a proper transfer does not follow (i.e., Wilson et al., 2002). Extant training literature has argued that learning transfer is a concern for organizations (Friedman and Ronen, 2015). *Learning transferability* has been a long-time researched topic in educational psychology (Hung, 2013). Adapting acquired knowledge in changed or modified forms in diverse situations to complete a given task or solve problems based on cognitive processes roughly defines “learning transfer” (Hung, 2013). Training evaluation and training effectiveness have drawn significant recognition in the literature (e.g., Broad and Newstrom, 1992; Kraiger, 2002; Noe and Colquitt, 2002; Holton, 2003). Although two terms, *training evaluation* and *training effectiveness*, are interchangeably used, they are independent of each other (Alvarez et al., 2004). *Training evaluation* means the appropriateness, relevance, and usefulness measured by the reaction to training content and design items. It is more of a “measurement technique” to check if the training programs meet the goals as planned (Alvarez et al., 2004). In contrast, *training effectiveness* investigates the variables that might affect training outcomes throughout the process (pre/during/post) and could be estimated by transfer measures and post-training attitudes (Alvarez et al., 2004). Several conceptual models (e.g., Tannenbaum et al., 1993; Holton, 1996) were suggested to measure training effectiveness. In summary, training evaluation is a methodological approach for measuring training outcomes, while the latter is a theoretical effort to understand the causalities.

However, the studies dealing with the various effects of variables and timewise training outcome comparison are limited (Grossman and Salas, 2011; Massenberg et al., 2017). As previously discussed, the evaluation of extended training intervention adopting technology should be able to (1) measure transfer performance adequately and (2) explain training effectiveness that could capture the states of trainees.

### Advancing the CIPP evaluation model

**FIGURE 2:**
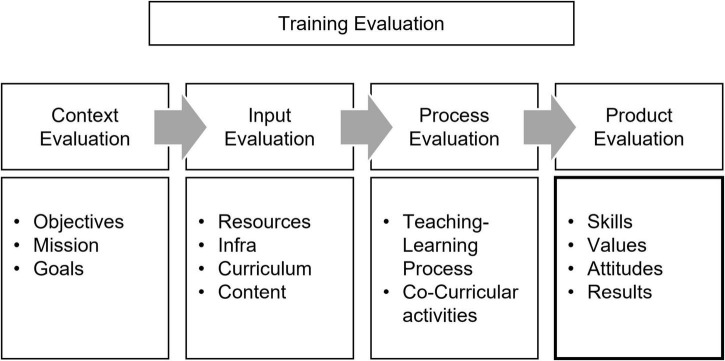
CIPP evaluation model.

Researchers have tried to extend the existing models to measure the training outcome in the literature, and the CIPP is one of the most frequently referenced models (e.g., Adedokun-Shittu and Shittu, 2013; Finney, 2019). CIPP is a comprehensive framework for evaluation aimed at long-term, sustainable interventions (Stufflebeam, 2004), standing for *context, input, process, and product* evaluation. CIPP reviews four areas of training (Figure 2). Context evaluation focuses on the overall objectives. Input evaluation concentrates on resources (infra, curriculum, content, and material), while process evaluation sees the teaching–learning process or other activities. The attitudinal and behavioral changes are regarded as the outcomes during the product evaluation. Generally, the CIPP is often sequentially applied following the orders of context–input–process–product assessment to confirm the accountability of a program and review the quality of each step (Stufflebeam, 2004). Using CIPP, the evaluators could grasp how well and effectively the training outcomes are achieved.

However, there are several limitations to the CIPP model. First, although it is a robust model to measure training outcomes, several situations would not allow smooth evaluation due to the complicated dynamics of stakeholders (Robinson, 2002; Tan et al., 2010). Second, it requires tremendous effort and resources, leading to slow evaluation. Third, CIPP is a framework for the evaluator, not the trainee, and might lack actual feedback from the trainees unless adequately applied (Tan et al., 2010). Finally, as pointed out in the previous section, the dynamic aspect of training “effectiveness” should be considered (i.e., Alvarez et al., 2004), including transfer measures and post-training attitudes. The CIPP model requires further updates and theoretical concerns to prove itself as a more robust framework.

This study suggests a new conceptual framework based on the CIPP model, attempting to close the theoretical gaps unveiled above. Then, the new framework collects and analyzes empirical data. The unique effectiveness model that (1) is more concise, (2) enables practical evaluation, (3) realizes trainee-centered evaluation, and (4) could measure learning transferability is presented in the following section.

## Methods

### Analytical procedure

The study includes the following analytical procedures. First, this study suggests a novel CIP-R model that overcomes the limitations of the existing CIPP model. Second, evaluation data for traditional/pure digital/hybrid training methods were collected from the sales training sessions administered in 2019 and 2020. Third, frequency analysis of the collected data and descriptive statistics for each variable are presented. Fourth, the three training methods are comparatively analyzed, and ANOVA confirms the statistically significant differences. Fifth, factors affecting the learning transfer are identified and compared by general linear model analysis. For all the statistical analysis, *the jamovi ver. 1.8.2* software (jamovi, 2021) was used.

### The CIP-R framework

The primary goal of this article was to analyze and compare the different modes of sales training enabled by digital technology and examine the factors affecting training effectiveness. As discussed in the “Advancing the CIPP evaluation model” section, the article adopted CIPP as the base framework and attempted to provide a new effectiveness model, further improving its limitations.

For decades, organizations have shifted from satisfaction to next-level constructs to measure training effectiveness (Mattox, 2013). Kirkpatrick (1998) argued that organizations should avoid using one simple “*satisfaction measure”* to measure training’s value. The rest of the stages in Kirkpatrick’s model focus on more critical aspects of learning transfer, which is training effectiveness (Mattox, 2013). Researchers such as Phillips (2012) and Brinkerhoff (2003) also advocated the necessity of “beyond satisfaction” measures in the post-training stages.

Satisfaction and loyalty (*recommendation*) are two concepts that have become crucial constructs in modern management (Kristensen and Eskildsen, 2011). The positive causal effect of satisfaction on loyalty has been proved in the literature (i.e., Tuu and Olsen, 2010). Tracking promoters propose organizations a compelling way to measure loyalty (Reichheld, 2003), and promoters who recommend services or products to others are loyal enthusiasts (Reichheld, 2006). This basic idea of focusing on recommendations is borrowed from the net promoter score (NPS) concept by Reichheld (2003). NPS measures the “recommendation” by the customers’ rating toward a simple question asking if they want to “recommend” the service or product they experienced to other close people such as friends, family, or colleagues, using a 0- to 10-point scale. The rating, which is believed to present loyalty, helps divide the respondents into three groups, namely, promoters (9–10), passives (7–8), and detractors (0–6). Considering recommendation factor in training is now commonplace across organizational units and utilized frequently as a valuable predictor and effectiveness measure for HR practices (Mattox, 2013) since it is *not the driver* but an *outcome of training*. Certainly, if trainees learn new skills and knowledge, they might want to apply the learning to their jobs and encourage others to do the same when successful. In this respect, *recommending a training program* could be a strong predictor of learning transfer.

**FIGURE 3:**
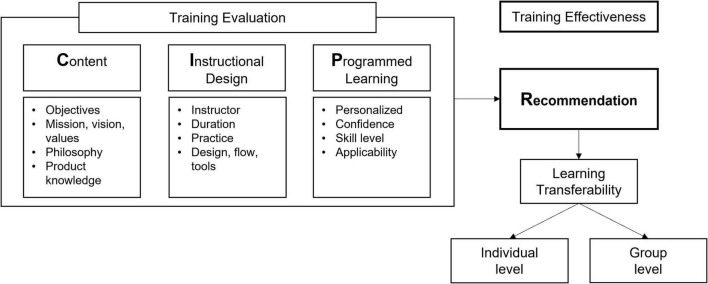
CIP-R effectiveness model.

Based on the above discussions, the existing CIPP model was reviewed and redesigned regarding the following two issues. First, the new framework should endorse digital training’s particular characteristics, enabling an apple-to-apple comparison. In this respect, the new framework excluded spatial factors to reflect the digital training’s characteristics and offline-only elements from the measurements. Second, ambiguous and almost-duplicative factors should be removed or simplified for clarity. For example, excessively detailed measures for context (e.g., objectives, goals) and input (e.g., content, curriculum) evaluation in CIPP may confuse the trainees while evaluating. As a result, a new training effectiveness framework, the CIP-R model, was presented (Figure 3). CIP-R is an acronym for content, instructional design, programmed learning, and recommendation. *Recommendation* measures loyalty. It was adopted for learning transferability toward individual and group levels, which is the key to training effectiveness. As discussed in the “Theoretical background” section, the C-I-P could be viewed as the training evaluation, and the R (recommendation) could be understood as a training effectiveness measurement.

### Data collection

As explained in the “Digital technology in a corporate learning context” section, the study takes the empirical data from STEP (sales consultant training and education program) of Company A, based in South Korea, an importer of global premium and mass automobile brands. Three HRD experts in automotive sales training and two Ph.D. researchers participated in the analytical procedure. Sales training data from March 2019 to October 2020 were used. Trainees assessed all training sessions through an online survey. To ensure unbiased evaluation, the survey did not ask for personal information. The collected survey results were sent to the firm’s training experience management system. The measurements of the CIP-R framework are shown in Table 1, and the 5-point Likert scale was applied to all items. STEP is a structured program designed to maximize the competence of sales consultants and aims to pursue customer delight through quality sales. The training is a 3-day, off-site, traditional classroom-type program. Due to the suspension of training during COVID-19, the company shifted to pure digital (from April to June 2020) and hybrid training (from July to October 2020) mixed online and offline.

**TABLE 1:** Measurements.

Category		Code	No.	Evaluation
Independent variable	Content	C_Obj	1	Objectives and goals of learning
		C_Mis	2	Mission, vision, values
		C_Phi	3	Sales and service philosophy
		C_Pro	4	Product knowledge
	Instructional Design	I_Lec	5	Lecturer and instructor engagement
		I_Dur	6	Duration of training
		I_Pra	7	Practice and participation
		I_Des	8	Design, flow and learning tools
	Programmed Learning	P_Per	9	Personalized learning
		P_Con	10	Confidence and overall competence
		P_Skl	11	Skill level for the job
		P_App	12	Applicability
Dependent variable	Recommendation	R_Rec	13	Recommendation (transferability)

All 5-point Likert scale.

In the traditional training conducted in 2019, 325 trainees participated in the 3-day session. Offline-based lectures, discussions, role-playing, and problem-solving sessions were implemented. A total of 140 sales executives participated in the pure digital training. Lectures and interactions were not provided, and the course depended on the learner’s self-directedness. Hybrid training was a well-blended version of offline and online. A total of 118 employees participated, and real-time live streaming sessions were provided. After the online session, instructors visited the learners’ offices to check their competencies and conducted role-play and problem-solving evaluations. First, the instructors handed out various scenarios, including multiple car purchase scenarios for customers with differentiated needs. Second, they evaluated each personnel based on structured measurement scales and checklists to assess sales consultants’ capabilities expected to be enhanced throughout the prior online learning sessions.

Although the three methods differed in their implementation, there was no significant difference in each course’s learning objectives and curriculum. The traditional method was the highest in terms of cost, the hybrid type was medium, and the pure digital training was the lowest. The details of the three training modes and frequency table are presented in Tables 2, 3. Brand: premium brand salespeople 59.2%, mass brand 40.8%. Grade: certified (42.5%), expert (33.3%), pioneer (23.5%), master (0.7%). Training methods: traditional (55.7%), pure digital (24.0%), and hybrid (20.2%).

**TABLE 2:** Comparison: traditional, pure digital, and hybrid training.

	Traditional	Pure digital	Hybrid
When	2019	2020 1H	2020 2H
Participants	325	140	118
Cost	High	Low	Mid
Duration	3 days	3 days	Flexible
Characteristics	100% offline	100% online	8H (online) + 8H (offline)
Program	Orientation	Orientation (0.5 day)	Orientation*
	Lecture-Interaction	Self-learning (2.5 days)	Lecture-Interaction*
	Role-playing	Role-playing (video)	Role-playing**
	Problem-solving	Problem-solving (paper)	Problem-solving**
	Evaluation-Certification	Evaluation-Certification	Evaluation-Certification**

*Online webinar and live streaming.

**Offline visit to each trainee’s site: personal instruction and evaluation.

**TABLE 3:** Frequency: brand, sales consultant grade, and training method.

Levels		Counts	%	Cumulative%
Brand	Premium	345	59.2	59.2
	Mass	238	40.8	100.0
Grade	Master	4	0.7	0.7
	Pioneer	137	23.5	24.2
	Expert	194	33.3	57.5
	Certified	248	42.5	100.0
Method	Hybrid	118	20.2	20.2
	Pure digital	140	24.0	44.2
	Traditional	325	55.7	100.0

*N* = 583.

## Results

### Outcome comparison of three training methods

To compare the effectiveness of the three training methodologies, the evaluation results by training participants were analyzed. A total of 583 data instances were used for the analysis. The basic summary statistics results are as follows (Table 4). The mean value of the items was 4.266–4.501. The item with the highest value was the “Sales and service philosophy” item in content evaluation, and the lowest item was the “Practice and participation” item in instructional design. The results indicate that the training participants were relatively satisfied with the training content delivering the firm’s philosophy and were less content with participatory and practical learning settings. The mean of all variables was 4.419. The average values of each dimension of content, instructional design, and programmed learning were 4.471, 4.386, and 4.409, respectively, and it was found that the content dimension received relatively higher ratings than the other three dimensions.

**TABLE 4:** Summary statistics.

	C_Obj	C_Mis	C_Phi	C_Pro	I_Lec	I_Dur	I_Pra	I_Des	P_Per	P_Con	P_Skl	P_App	R_Rec
Mean	4.432	4.489	4.501	4.463	4.532	4.410	4.266	4.334	4.345	4.401	4.400	4.491	4.383
SD	0.719	0.708	0.715	0.745	0.734	0.813	0.898	0.812	0.759	0.751	0.748	0.668	0.766
Variance	0.517	0.501	0.512	0.555	0.538	0.662	0.807	0.659	0.577	0.564	0.560	0.446	0.587
Skewness	−1.138	−1.404	−1.360	−1.337	−1.787	−1.380	−1.147	−1.146	−1.071	−1.347	−1.197	−1.267	−1.256
Kurtosis	1.010	2.157	1.504	1.582	3.763	1.715	0.965	0.962	0.953	2.342	1.397	1.806	1.839

*N* = 583.

All variable mean = 4.419.

Mean for content = 4.471, mean for instructional design = 4.386, mean for programmed learning = 4.409.

Then, all variables were divided by three training methodologies, and group statistics were derived (Table 5). By comparing the mean value of each item, it was confirmed in which area the differences in the training methodology developed significantly. As for the “recommendation” item that indicates training effectiveness, the traditional method appeared to be the highest (4.523), and the pure digital method was the lowest (4.093), while the hybrid method was in the middle (4.339). In the first half of 2020, when all the training interventions shifted from traditional to pure digital due to COVID-19, the item where the trainees’ rating decreased the most was the “Lecturer and instructor engagement” in the instructional design dimension (traditional = 4.686, pure digital = 4.086, difference = −0.600). The second highly impacted item was the “Duration of training” in the same dimension, showing a steep decrease (traditional = 4.560, pure digital = 4.114, difference = −0.446). The item that showed the slightest change was the “applicability” item in the programmed learning dimension (traditional = 4.520, pure digital = 4.386, difference = −0.134). In the second half of 2020, training shifted from pure digital to the hybrid method, and the overall ratings were improved. The “Lecturer and instructor engagement” item in the instructional design dimension indicated the most improvement (pure digital = 4.086, hybrid = 4.636, difference = + 0.550). The next most improved area was the “Practice and participation” in the instructional design dimension (pure digital = 3.971, hybrid = 4.263, difference = + 0.292). The item with the most negligible difference was “Confidence and overall competence” in the programmed learning dimension (pure digital = 4.286, hybrid = 4.364, difference = + 0.078).

**TABLE 5:** Group descriptive by training method.

	Method	N	Mean	SD	SE
R_Rec	Hybrid	118	4.339	0.808	0.074
	Pure digital	140	4.093	0.839	0.071
	Traditional	325	4.523	0.678	0.038
C_Obj	Hybrid	118	4.466	0.712	0.066
	Pure digital	140	4.200	0.850	0.072
	Traditional	325	4.520	0.636	0.035
C_Mis	Hybrid	118	4.500	0.725	0.067
	Pure digital	140	4.243	0.839	0.071
	Traditional	325	4.591	0.610	0.034
C_Phi	Hybrid	118	4.508	0.713	0.066
	Pure digital	140	4.307	0.813	0.069
	Traditional	325	4.582	0.655	0.036
C_Pro	Hybrid	118	4.373	0.845	0.078
	Pure digital	140	4.279	0.823	0.070
	Traditional	324	4.577	0.647	0.036
I_Lec	Hybrid	118	4.636	0.565	0.052
	Pure digital	140	4.086	0.985	0.083
	Traditional	325	4.686	0.567	0.031
I_Dur	Hybrid	118	4.347	0.881	0.081
	Pure digital	140	4.114	0.922	0.078
	Traditional	325	4.560	0.694	0.038
I_Pra	Hybrid	118	4.263	0.862	0.079
	Pure digital	140	3.971	1.038	0.088
	Traditional	325	4.394	0.816	0.045
I_Des	Hybrid	118	4.322	0.815	0.075
	Pure digital	140	4.121	0.869	0.073
	Traditional	325	4.431	0.769	0.043
P_Per	Hybrid	118	4.297	0.820	0.075
	Pure digital	140	4.171	0.758	0.064
	Traditional	325	4.437	0.724	0.040
P_Con	Hybrid	118	4.364	0.813	0.075
	Pure digital	140	4.286	0.752	0.064
	Traditional	325	4.465	0.722	0.040
P_Skl	Hybrid	118	4.415	0.766	0.071
	Pure digital	140	4.221	0.805	0.068
	Traditional	325	4.471	0.705	0.039
P_App	Hybrid	118	4.534	0.650	0.060
	Pure digital	140	4.386	0.674	0.057
	Traditional	325	4.520	0.669	0.037

Finally, an ANOVA was conducted for each variable. The result displayed that there is a statistically significant difference stemming from training methodology in all items except for two, “Confidence and overall competence” (*F* = 3.009, *p* > 0.05) and “Applicability” (*F* = 2.264, *p* > 0.05) in the programmed learning dimension (Table 6). Additionally, *post hoc* test results were provided to check the significant mean differences found in the variables based on the training methodology (Table 7).

**TABLE 6:** One-way ANOVA.

	F	df1	df2	p
R_Rec	14.930	2	239.878	< 0.001
P_Per	6.457	2	251.102	0.002
P_Con	3.009	2	251.663	0.051
P_Skl	5.036	2	249.380	0.007
P_App	2.264	2	263.306	0.106
I_Lec	22.770	2	239.014	< 0.001
I_Dur	14.115	2	233.032	< 0.001
I_Pra	9.240	2	246.319	< 0.001
I_Des	6.683	2	251.586	0.001
C_Obj	7.979	2	240.342	< 0.001
C_Mis	9.848	2	235.396	< 0.001
C_Phi	6.234	2	245.458	0.002
C_Pro	8.663	2	233.468	< 0.001

*N* = 583.

ANOVA: Welch’s.

**TABLE 7:** *Post hoc* tests.

		Mean difference	
		Pure digital	Traditional
R_Rec	Hybrid	0.246*	−0.184
	Pure digital	—	−0.430***
P_Per	Hybrid	0.125	−0.140
	Pure digital	—	−0.265**
P_Con	Hybrid	0.079	−0.100
	Pure digital	—	−0.179*
P_Skl	Hybrid	0.194	−0.056
	Pure digital	—	−0.249**
P_App	Hybrid	0.148	0.014
	Pure digital	—	−0.134
I_Lec	Hybrid	0.550***	−0.051
	Pure digital	—	−0.600***
I_Dur	Hybrid	0.233	−0.213*
	Pure digital	—	−0.446***
I_Pra	Hybrid	0.291*	−0.131
	Pure digital	—	−0.422***
I_Des	Hybrid	0.201	−0.109
	Pure digital	—	−0.309***
C_Obj	Hybrid	0.266*	−0.054
	Pure digital	—	−0.320***
C_Mis	Hybrid	0.257*	−0.091
	Pure digital	—	−0.348***
C_Phi	Hybrid	0.201	−0.073
	Pure digital	—	−0.274**
C_Pro	Hybrid	0.094	−0.204*
	Pure digital	—	−0.299***

*Post hoc* test: Games–Howell.

*p < 0.05, **p < 0.01, ***p < 0.001.

### Factors affecting training effectiveness

Then, the study verified the results of the ANOVA omnibus test and the fixed-effects parameter estimates result (Gallucci, 2019) to confirm the variables’ effect sizes on training effectiveness and the coefficient for each term (Tables 8, 9). The three sales training methods—traditional, pure digital, and hybrid—showed statistically significant differences (*F* = 4.882, *p* < 0.01). The result confirmed a statistically significant difference between the pure digital method and the traditional training effectiveness (*B* = −0.121, *p* < 0.01). The difference between the hybrid training and the pure digital method was not statistically significant (*B* = 0.040, *p* > 0.05). The existence of five factors affecting the training effectiveness (recommendation) was confirmed. “Lecturer and instructor engagement” (*F* = 17.783, *B* = 0.134, *p* < 0.001), “Duration of training” (*F* = 27.798, *B* = 0.162, *p* < 0.001) in instructional design dimension and “Personalized learning” (*F* = 9.231, *B* = 0.121, *p* < 0.01), “Confidence and overall competence” (*F* = 52.304, *B* = 0.330, *p* < 0.001), and “Skill level for the job” (*F* = 11.221, *B* = 0.168, *p* < 0.001) in programmed learning dimension confirmed the clear difference by methodology and appeared to affect the dependent variable in a statistically significant way.

**TABLE 8:** ANOVA omnibus test.

	SS	df	F	p	η^2^	η^2^p	ω^2^p	ε^2^p
Model	19.128	14	128.67	< 0.001	0.761	0.761	0.754	0.755
Method	1.408	2	4.882	0.008	0.054	0.185	0.178	0.182
C_Obj	0.052	1	0.362	0.548	1.41E-04	0.001	−0.001	−0.001
C_Mis	0.029	1	0.204	0.651	4.04E-05	0.000	−0.002	−0.002
C_Phi	0.074	1	0.512	0.475	1.14E-04	0.000	−0.001	−0.001
C_Pro	0.044	1	0.306	0.581	1.29E-04	0.001	−0.001	−0.001
I_Lec	2.565	1	17.783	< 0.001	0.018	0.070	0.067	0.068
I_Dur	4.009	1	27.798	< 0.001	0.019	0.075	0.071	0.073
I_Pra	0.002	1	0.013	0.908	5.24E-05	0.000	−0.002	−0.002
I_Des	0.378	1	2.618	0.106	0.001	0.006	0.004	0.004
P_Per	1.331	1	9.231	0.002	0.546	0.695	0.689	0.695
P_Con	7.543	1	52.304	< 0.001	0.106	0.306	0.300	0.305
P_Skl	1.618	1	11.221	< 0.001	0.012	0.047	0.044	0.045
P_App	0.075	1	0.521	0.471	0.004	0.015	0.013	0.014
Residuals	81.77	567						
Total	101.037	582						

Estimate: linear model fit by OLS.

Dependent variable: recommendation.

*R*^2^: 0.761.

Adj. *R*^2^: 0.755.

**TABLE 9:** Fixed-effects parameter estimates.

Names	Effect	Estimate	SE	95% Confidence interval		β	df	t	p
				Lower	Upper				
(Intercept)	(Intercept)	−0.122	0.125	−0.367	0.122	0.000	567	−0.981	0.327
Method1	Hybrid—Pure digital	0.040	0.050	−0.058	0.138	0.052	567	0.799	0.425
Method2	Pure digital—Traditional	−0.121	0.042	−0.203	−0.039	−0.158	567	−2.893	0.004
C_Obj	Objectives and goals of learning	0.026	0.044	−0.060	0.113	0.025	567	0.602	0.548
C_Mis	Mission, vision, values	−0.020	0.044	−0.106	0.067	−0.018	567	−0.452	0.651
C_Phi	Sales and service philosophy	0.033	0.046	−0.057	0.123	0.031	567	0.715	0.475
C_Pro	Product knowledge	−0.022	0.039	−0.099	0.055	−0.021	567	−0.553	0.581
I_Lec	Lecturer and instructor engagement	0.134	0.032	0.071	0.196	0.128	567	4.217	< 0.001
I_Dur	Duration of training	0.162	0.031	0.101	0.222	0.171	567	5.272	< 0.001
I_Pra	Practice and participation	−0.003	0.028	−0.059	0.052	−0.004	567	−0.115	0.908
I_Des	Design, flow and learning tools	0.055	0.034	−0.012	0.121	0.058	567	1.618	0.106
P_Per	Personalized learning	0.121	0.040	0.043	0.199	0.120	567	3.038	0.002
P_Con	Confidence and overall competence	0.330	0.046	0.240	0.419	0.323	567	7.232	< 0.001
P_Skl	Skill level for job	0.168	0.050	0.070	0.267	0.164	567	3.350	< 0.001
P_App	Applicability	0.033	0.045	−0.056	0.121	0.028	567	0.721	0.471

Estimate: linear model fit by OLS.

Dependent variable: recommendation.

R^2^: 0.761.

Adj. *R*^2^: 0.755.

**FIGURE 4:**
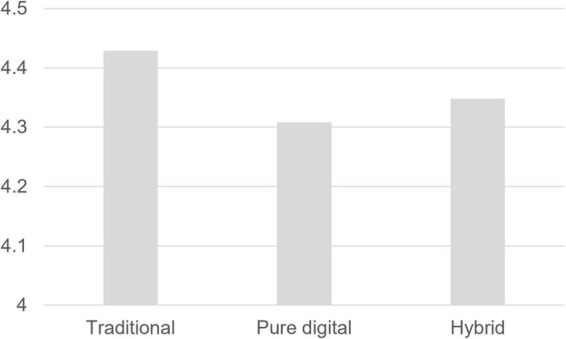
Training effectiveness: traditional, pure digital, and hybrid.

Finally, *estimated marginal means* of the dependent variable were calculated and compared for each training method to compare training effectiveness, reflecting the influence of each term included in the model. The result indicated that the traditional method has the highest effectiveness (mean = 4.429). The effectiveness decreased in the pure digital method (mean = 4.308) and then recovered after shifting to the hybrid training method that blended both (mean = 4.348). The result is presented in Table 10 and Figure 4.

**TABLE 10:** Estimated marginal means comparison.

Method	Mean	SE	df	95% Confidence interval	
				Lower	Upper
Hybrid	4.348	0.036	567	4.278	4.417
Pure digital	4.308	0.034	567	4.240	4.375
Traditional	4.429	0.022	567	4.386	4.471

Estimated means are estimated keeping constant other effects in the model to the mean. Variable: recommendation.

## Discussion

This study suggested a novel CIP-R training effectiveness framework to analyze the difference in training outcomes derived from the adoption of digital technology and verify which factors influence training effectiveness. The findings of the study are summarized as follows. First, the results of this study confirmed the effectiveness of *blended learning*, the *hybrid method* (Table 10). The overall sales training effectiveness fell as the firm shifted from the traditional to the pure digital (pure digital—traditional, mean difference = –0.121). However, the effectiveness was recovered with the hybrid method (hybrid—pure digital, mean difference = 0.040). Pure digital training required a complete self-directedness of participants and showed strength in terms of time and cost but had the lowest effectiveness. Blending the advantages of digital and offline could enhance learning effectiveness (Mantyla, 2001) and under today’s digital transformation, advancing traditional methods by technology-based learning (e.g., Driscoll, 2002; Graham, 2006) might be a suitable way of talent development (i.e., Liu et al., 2020).

Second, the findings confirmed the importance of human factors in training. The variables that caused visible differences in training effectiveness were primarily related to human involvement. The score of “Lecturer and instructor engagement” in the instructional design dimension decreased the most (pure digital—traditional, mean difference = –0.600) and then recovered to the maximum (hybrid—pure digital, mean difference = 0.550). Also, the “Practice and participation” item of the same dimension showed the second-largest increase (hybrid—pure digital, mean difference = 0.291). In the “Duration of training” item, which evaluates the appropriateness of learning time, the second-largest decrease was confirmed (pure digital—traditional, mean difference = −0.446). This empirical evidence confirms the claims that social interactions with colleagues in a digital learning setting play a crucial role in trainee engagement (i.e., Kim, 2021). Although traditional and pure digital training had the same learning duration (3 days), the perceived duration of online self-directed training might have been felt loosely. In this respect, social relationships might have to be regarded as a substantial factor in building a proper learning environment.

In contrast, the ANOVA result displayed that there were no statistically significant differences by training method in the “Confidence and overall competence” and “Applicability” items of the programmed learning dimension (Table 6). Despite the changed training methodologies, the actual content changed little; accordingly, the training program’s perceived value (valence) may not have changed. However, a statistically significant difference was found in the same dimension, in the “Personalized learning” and “Skill level for the job” items. This finding may support the assumption that the human factors caused a difference positively regarding training effectiveness. In fact, in hybrid training, instructor–trainee interaction was strengthened, and practice and feedback sessions were provided through field visits.

Third, businesses must understand the role of efficacy and self-determination. Factors that influenced the learning transferability were mostly variables that define how the learning is delivered to the participants and variables related to trainees’ benefits (two variables in the instructional design dimension and three in the programmed learning dimension). In fact, in this study, the four variables in the content dimension were found to have little effect on learning transfer (Table 9). If personalized learning is provided, trainees could feel confident in their knowledge and skills, have higher expectations for improved competency, and develop actual work performance based on learning transfer (Colquitt et al., 2000). It can be explained by the *self-efficacy* of individuals and groups (i.e., Bandura, 2000).

Moreover, it can be expected that *self-determined learning* will occur through programs that provide a sense of self-efficacy (i.e., Ryan and Deci, 2000). As the training program shifted to hybrid, trainees appeared to have found more value. If intrinsic motivation increases, behaviors to disseminate knowledge and skills acquired during training to peers would increase. Based on empirical evidence, extant literature argued that intrinsic motivators such as self-efficacy in knowledge/skill and enjoyment of supporting others might expedite the transfer of acquired competencies, enhancing the upper-level or group performance (e.g., Lin, 2007; Wen and Lin, 2014; Na-Nan and Sanamthong, 2020). Consequently, it can be assumed that understanding group psychology and reflecting it on talent management might lead to higher workforce performance.

## Theoretical and managerial implications

The study provides theoretical implications as follows. First, this study suggested the CIP-R framework, a novel effectiveness model that overcomes the limitations of the CIPP model. In particular, the study differentiated itself from the existing models by defining the trainee’s intention to recommend as a measurement for future learning transferability. The studies that provided empirical evidence of the effects of training variables and learning transferability as training outcomes are limited in the literature (Grossman and Salas, 2011; Massenberg et al., 2017). The CIP-R framework and the empirical evidence presented in this study are expected to contribute to existing domain knowledge and theoretical expansion. Second, by demonstrating the effects of digital technology in training, the study drew academic interest in sub-variables in each dimension. Moreover, the study calls for scholarly attention to organizational psychology theories by shedding light on individual/group efficacy (i.e., Bandura, 2000) and self-determination theories (i.e., Ryan and Deci, 2000), searching for a structural mechanism leading to learning transferability. The findings of this study present a perspective on how existing theories should change and be applied as digital technology expands.

The managerial implications of the study are as follows. First, the importance of digital technology acceptance should be reviewed from the talent development perspective. From a business standpoint, it is unimaginable to return to the traditional ways of developing people. HR professionals should consider development programs and provide flexible, personalized learning online (Li and Lalani, 2020). This study attempted both a blended learning format and an entirely online program. The findings of this research could be a practical example for future HRD professionals. Second, the study devised a practical and efficient framework for workforce development. Constant upskilling would be critical for organizations. Businesses need a useful and ready-to-use recipe for their people development initiatives. Existing effectiveness models are resource-taking and inadequate to measure the dynamic aspects of the learning or unreasonably focus on checking post-training changes (e.g., Robinson, 2002; Alvarez et al., 2004; Tan et al., 2010). Sometimes such academic interests fail to meet the actual needs of the business managers (Mattox, 2013). This study proposed the CIP-R framework as an alternative that efficiently measures learning transferability. It would enable practitioners to explore new opportunities to evaluate and improve training at a lower cost.

## Limitations and future research

This study has several limitations. First, it is difficult to generalize the research findings since the study was conducted in a specific context, such as the region and profession. It remains uncertain that the findings of this study could be applied to other cultures or workplace environments. Therefore, further studies are required to test the causalities between variables. Future researchers are invited to conduct repeated research with different subjects and contexts. Second, the conceptual framework presented in this study requires additional validation. It is needed to examine the utility of the CIP-R model through additional empirical studies. The replicated usage can strengthen the model’s validity, and its theoretical/managerial value could be proved. The critical views from subsequent researchers, model adjustments, and measurement changes are also considered meaningful. The study used statistical regression to examine causal relationships and effect sizes. If enough data instances are secured, using the recently preferred machine learning (ML) method could be valuable (Kim et al., 2021). Using ML techniques, non-linear, hidden relationships might be discovered. Third, the study did not track trainees’ post-training behavioral changes or the financial results generated. However, training is not the only driver for organizational performance improvement. Hence, it is recommended to study other related variables, such as the working environment, motivation, and exchange relationships in the workplace, which might be related to sustaining the post-training performance.

## Data availability statement

The raw data supporting the conclusions of this article will be made available by the authors, without undue reservation.

## Ethics statement

Ethical review and approval was not required for the study on human participants in accordance with the local legislation and institutional requirements. Written informed consent from the patients/participants was not required to participate in this study in accordance with the national legislation and the institutional requirements.

## Author contributions

SK was responsible for the conceptualization, data collection, methodology, analytical procedures (quantitative), writing – original draft, review and editing.

## Conflict of interest

The author declares that the research was conducted in the absence of any commercial or financial relationships that could be construed as a potential conflict of interest.

## Publisher’s note

All claims expressed in this article are solely those of the authors and do not necessarily represent those of their affiliated organizations, or those of the publisher, the editors and the reviewers. Any product that may be evaluated in this article, or claim that may be made by its manufacturer, is not guaranteed or endorsed by the publisher.
